# Serological survey of neutralizing antibodies to eight major enteroviruses among healthy population

**DOI:** 10.1038/s41426-017-0003-z

**Published:** 2018-01-10

**Authors:** Rui Zhu, Tong Cheng, Zhichao Yin, Dongxiao Liu, Longfa Xu, Yongchao Li, Wei Wang, Jian Liu, Yuqiong Que, Xiangzhong Ye, Qiyi Tang, Qinjian Zhao, Shengxiang Ge, Shuizhen He, Ningshao Xia

**Affiliations:** 1 State Key Laboratory of Molecular Vaccinology and Molecular Diagnostics, School of Life Sciences, School of Public Health, Xiamen University, Xiamen, 361102 China; 2 Beijing Wantai Biological Pharmacy Enterprise, Beijing, 102206 China; 3 Department of Microbiology, Howard University College of Medicine, Washington, DC 20059 USA; 4 Xiamen Center for Disease Control and Prevention, Xiamen, 361012 China

## Abstract

Human enteroviruses (EVs) are the most common causative agents infecting human, causing many harmful diseases, such as hand, foot, and mouth disease (HFMD), herpangina (HA), myocarditis, encephalitis, and aseptic meningitis. EV-related diseases pose a serious worldwide threat to public health. To gain comprehensive insight into the seroepidemiology of major prevalent EVs in humans, we firstly performed a serological survey for neutralizing antibodies (nAbs) against Enterovirus A71 (EV-A71), Coxsackie virus A16 (CV-A16), Coxsackie virus A6 (CV-A6), Coxsackie virus A10 (CV-A10), Coxsackie virus B3 (CV-B3), Coxsackie virus B5 (CV-B5), Echovirus 25 (ECHO25), and Echovirus 30 (ECHO30) among the healthy population in Xiamen City in 2016, using micro-neutralization assay. A total of 515 subjects aged 5 months to 83 years were recruited by stratified random sampling. Most major human EVs are widely circulated in Xiamen City and usually infect infants and children. The overall seroprevalence of these eight EVs were ranged from 14.4% to 42.7%, and most of them increased with age and subsequently reached a plateau. The co-existence of nAbs against various EVs are common among people ≥ 7 years of age, due to the alternate infections or co-infections with different serotypes of EVs, while most children were negative for nAb against EVs, especially those < 1 year of age. This is the first report detailing the seroepidemiology of eight prevalent EVs in the same population, which provides scientific data supporting further studies on the improvement of EV-related disease prevention and control.

## Introduction

Human enteroviruses (EVs), the most common pathogens infecting humans, are grouped into four species (A–D) based on genotype, including polioviruses, coxsackie viruses, echoviruses, and other EVs^1^. Human EV infections cause a wide range of diseases with varying degrees of severity, such as hand, foot, and mouth disease (HFMD), herpangina (HA), myocarditis, encephalitis, aseptic meningitis, gastroenteritis, non-specific febrile illness and so on^2^. The emergence and transmission of human EV-related diseases result in increased economic and public health burdens.

The human EV-A species is responsible for the majority of HFMD, which is a highly contagious disease that mainly affects infants and young children. Most patients recover fully in 7 to 10 days, but some develop severe central nervous system (CNS) complications and even death^3^. Enterovirus A71 (EV-A71), Coxsackie virus A16 (CV-A16), Coxsackie virus A6 (CV-A6), Coxsackie virus A10 (CV-A10) are the major pathogens of HFMD, which have caused large outbreaks worldwide, leading to significant morbidity and mortality^4^. The human EV-B species also causes some sporadic HFMD cases^8, 9^, but is more commonly associated with serious diseases, including myocarditis, aseptic meningitis, encephalitis, and hepatitis, which are more representative pathogen of Coxsackie virus B3 (CV-B3)^10^, Coxsackie virus B5 (CV-B5)^11^, Echovirus 30 (ECHO30)^12, 13^, and Echovirus 25 (ECHO25)^14^. Moreover, the population of echovirus infections involve both children and adults^15^, and ECHO30 is frequently isolated in EV surveillance in Europe and North America^16^.

EV species diversity can be tied to the notable phenotypic variability. Co-infection and co-circulation of various viruses have been commonly reported, especially in HFMD outbreaks^17–19^. Such co-infection in individuals and co-circulation in epidemics not only exaggerated the severity of disease, but led to the emergence of genomic recombinant strains^20, 21^. However, cross-neutralizing antibodies against different EVs were not been observed, and no specific therapeutic drug or multivalent vaccine was available. When a human is infected with viruses, nAbs typically play a central role in their defense. Clarification of the nAb levels among different age groups is essential for understanding the history of previous infection and herd-immunity, and improves the prevention and control viral infections. Currently, the traditional micro-neutralization assay remains the “gold standard” detection method for nAbs, although this method is cumbersome and time-consuming. For now, only a few serum surveys on human EVs have been conducted using this method in Brazil, Singapore, Germany and a few provinces in China, mostly about EV-A71 and CV-A16 in children^22^. There has been no report of serological survey on more than three human EVs in a cohort, and the seroepidemiology researches of CV-B5, ECHO25, and ECHO30 were not reported.

In our previous study, we have conducted some researches on infectious cDNA clones^14, 27^, virus structures^28^, animal models^29, 30^, and vaccines^31–]^ of various human EVs. Herein, in order to gain a more comprehensive insight into the seroepidemiology of human EVs for supporting the development of broad-spectrum therapeutic drug and multivalent vaccine against human EVs, we carried out a serological study of EV-A71, CV-A16, CV-A6, CV-A10, CV-B3, CV-B5, ECHO25, and ECHO30 in a large sample of children and adults in Xiamen City.

## Materials and methods

### Study subjects and serum samples

Healthy subjects participated in immune status surveillance of infectious diseases at the Xiamen City Center for Disease Control and Prevention (CDC) in the Fujian Province in China in 2016. A total of 515 subjects were recruited by stratified random sampling in a cross-sectional epidemiological survey according to districts and age groups (Table 1). All participants had no sign of disease at the time of sample collection. All serum samples were divided and stored at −20 °C and inactivated at 56 °C for 30 min before testing. Written consent was obtained from each participant. The study was approved by the Ethics Committees of the Fujian Provincial CDC and the Research Ethics Review Committee of Xiamen University.

**Table 1:** Demographic characteristics of subjects in serological survey

Age (years)	Age (mean ± SD)	Male *n* (%)	Female *n* (%)	Total *n* (%)
<1	0.7 ± 0.2	20 (44.4)	25 (55.6)	45 (8.74)
1–3	1.7 ± 0.7	35 (52.2)	32 (47.8)	67 (13.01)
4–6	4.7 ± 0.9	49 (51.0)	47 (49.0)	96 (18.64)
7–19	13.1 ± 4.3	45 (52.3)	41 (47.7)	86 (16.7)
20–39	29.9 ± 5.7	30 (44.8)	37 (55.2)	67 (13.01)
40–59	49.3 ± 5.7	43 (50)	43 (50)	86 (16.7)
≥60	66.3 ± 5.8	33 (48.5)	35 (51.5)	68 (13.2)
Total		255 (49.5)	260 (50.5)	515

All healthy subjects were recruited from an status surveillance of infection diseases at the Xiamen City Center for Disease Control and Prevention in the Fujian Province in China in 2016.

### Micro-neutralization test

**Fig. 1:**
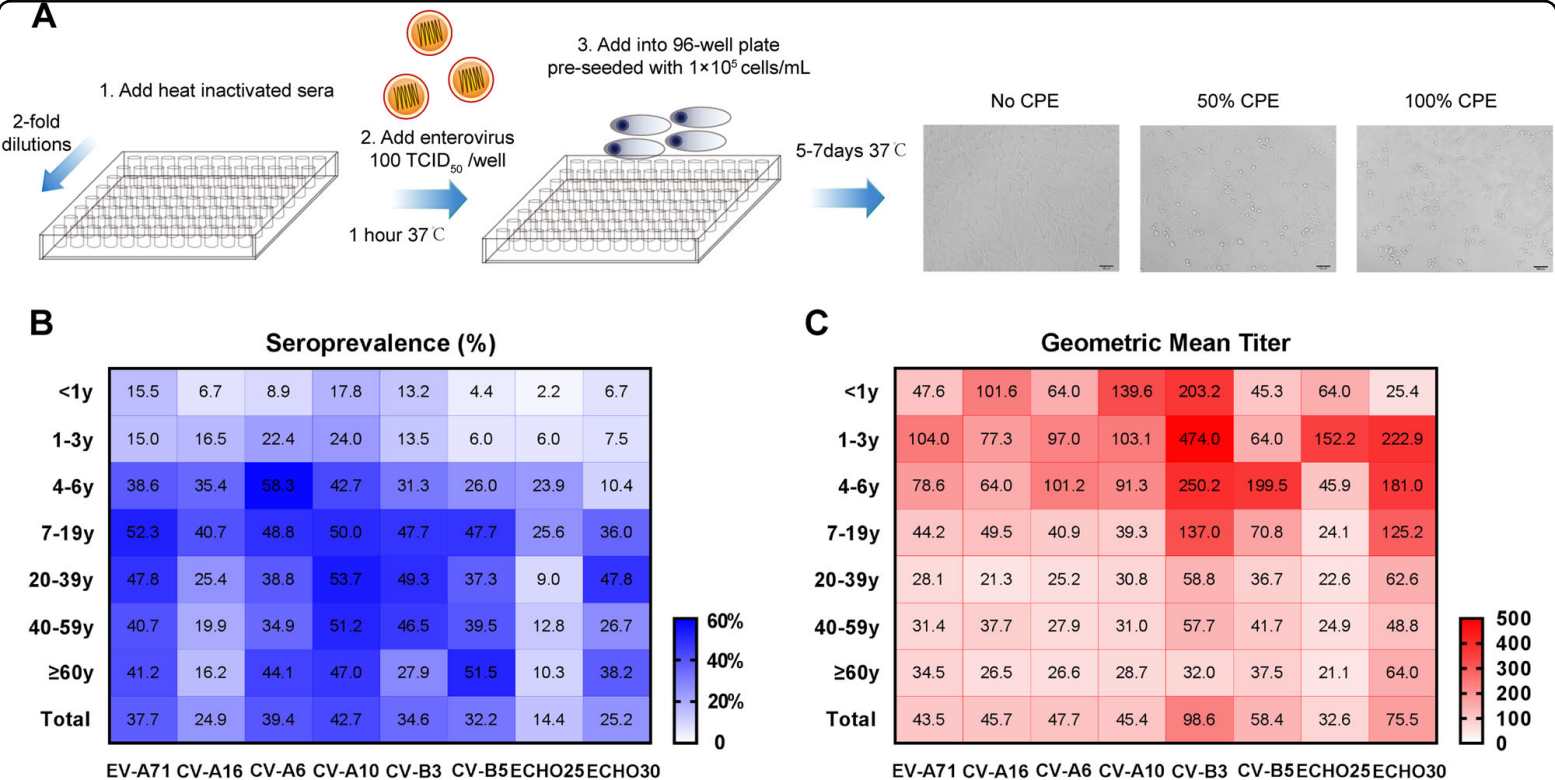
Overview of micro-neutralization assay (**a)** and the age distributions of seroprevalence (**b)** and GMT **(c)** against eight types of EVs. The main steps of the micro-neutralization assay are shown in **a**. After step 3, the diluted heat inactivated sera, enterovirus and RD cells were incubated at 37 °C or 33°C (EV-D68) for 5 to 7 days. The detection of CPE was observed by microscope. The neutralizing titer was defined as the highest dilution that exhibited > 50% neutralization of the well. Heat maps illustrating the age distributions of seroprevalence (**b**) and GMT (**c**) against eight types of EVs (EV-A71, CV-A16, CV-A6, and CV-A10 are belong to EV-A species; CV-B3, CV-B5, ECHO25, and ECHO30 are belong to EV-B species). A higher seroprevalence or GMT is shown as a darker red or blue color. The values of seroprevalence and GMT are given in the map

In this study, EV-A71^31^, CV-A16^34^, CV-A6^27^, CV-A10^30^, CV-B3^35^, CV-B5, ECHO25^14^, and ECHO30 (Table 2) were used to quantify nAbs by micro-neutralization assay. These viruses were propagated in rhabdomyosarcoma (RD) cells (ATCC® CCL-136™) in our laboratory and the viral infectivity titers were determined as previously described^31^. An overview of micro-neutralization assay is shown in Fig. 1a. In brief, serum samples were diluted serially twofold from 1:16 to 1:2048 and incubated with an equal volume of virus at 100TCID_50_ at 37 °C for 1 h. The mixtures were then added into 96-well plates which were pre-seeded with 1 × 10^4^ cells/mL and incubated at 37 °C or 33°C (EV-D68) for 5 to 7 days. Each batch of tests included cell control, positive serum control, and negative serum control. A positive serum sample of known antibody titer ( ≥ 1:128) that was from mice immunized with one virus for its corresponding experimental. A negative serum sample was from unimmunized mice. All serum samples were tested in duplicate. The cytopathic effect (CPE) was observed by microscopy. The neutralizing titer was defined as the highest dilution that exhibited > 50% neutralization of the well. A neutralizing titer of ≥ 1:16 was considered as a threshold for positivity^36, 37^.

**Table 2:** The information on enterovirus strains used in the CPE assay

Virus strain^a^	Origin	Year	Genebank No.	Ref.
EV-A71/ C4	Jiangsu CDC	2008	FJ600325	Xu et al.^30^
CV-A16/190	National Taiwan University	2007	JF420555	Hou et al.^33^
CV-A6/141	Infectious cDNA clone	2007	KR706309	Yang et al.^27^
CV-A10	Xiamen CDC	2014	KY012321	Li et al.^29^
CV-B3/2035	Xiamen CDC	2008	JQ042700	Yang et al.^34^
CV-B5	Xiamen University	2009	JQ042699	/
ECHO25	Infectious cDNA clone	2013	KP099941	Hou et al.^14^
ECHO30	Xiamen University	2016	MF177223	/

^a^The EV-A71/C4 strain was kindly provided by Jiangsu CDC; the CV-A16/190 strain was kindly provided by National Taiwan University; the CV-A10 and CV-B3 strains were kindly provided by Xiamen CDC; the CV-A6/141 and ECHO25 strains were infectious cDNA clones constructed by our laboratory; the CV-B5 and ECHO30 strains were isolated by our laboratory

### Statistical analysis

The seroprevalence, neutralizing titer and geometric mean titer (GMT) were calculated. The titers beyond 1:2048 were assigned 1:2048. The chi-squared test or Mann–Whitney test were, respectively, used to test the significant differences in seroprevalence or GMTs between groups. Statistical analyses were performed using SPSS version 13.0 software (SPSS Inc., Chicago, IL, USA). The 95% confidence intervals (CI) for seroprevalence were calculated according to Wilson’s method, using the VassarStats website for statistical computation^37^. *P*-values of < 0.05 were considered statistically significant.

## Results

### Overall seroprevalence and GMT of different types of EVs

A total of 515 subjects aged between 5 months and 83 years (~50.5% female) were tested for nAbs against EVs (Table 1). The overall seroprevalence of eight EVs were varied and ranged from 14.4% to 42.7% (Fig. 1b). For EV-A species, the seroprevalence of CV-A10, CV-A6, and EV-A71 were 42.7% (95% CI: 38.5–47.0), 39.4% (95% CI: 35.3–43.7), and 37.7% (95% CI: 33.6–42.9), which were significantly higher than CV-A16 at 24.9% (95% CI: 21.3–28.8, *P* < 0.001), while there were no significant differences in the GMTs of nAbs among them (Fig. 1b, c). For EV-B species, the seroprevalence of CV-B3, CV-B5, and ECHO30 were between 25.2% and 34.6%, which were higher than ECHO25 (*P* < 0.05) (Fig. 1b). The GMTs of nAbs against CV-B3 and ECHO30 were higher at 98.6 and 75.5 (Fig. 1c). The overall seroprevalence and GMT of nAbs against ECHO25 were the lowest, with only 74 positive samples among 515 subjects, and the overall GMT was 1:32.6 (Fig. 1b, c).

### The seroprevalence and GMT of different types of EVs in age groups

The analysis of the different age groups revealed that the seroprevalence of EV-A71, CV-A6, CV-A10, CV-B3, CV-B5, and ECHO30 were increased with age, ranging from 4.4–17.8% in infants to 6.0–24.0% in toddlers to 10.4–58.3% in pre-schoolers, and a plateau was reached in the age group of 7 to 19 years and kept at a steady state subsequently, with an approximately 30 to 50% positive rate (Fig. 1b, Supplementary Fig. S1A, C-F, H). However, the seroprevalence of CV-A16 was found to be lower in most age groups among EV-A species (Fig. 1b, Supplementary Fig. S1B) and so did ECHO25 among EV-B species in this research (Fig. 1b, Supplementary Fig. S1G). In addition, the seroprevalence of CV-A16 and ECHO25 were also increased with age, but declined from their peaks of the 4 to 6 and 7 to 19 age groups (Fig. 1b, Supplementary Fig. S1B, G).

The GMT of nAbs against EVs in different age groups revealed that children and adolescents showed higher nAb titers than others in all eight EVs (Fig. 1c). To further analyze the immune response, nAb titers were defined into four ranges:  < 1:16 (no), 1:16–1:1:32 (low), 1:64–1:256 (moderate), and 1:512–1:2048 (high). For EV-A species, most children presented with moderate titer levels, whereas individuals aged > 7 years presented with low titer levels, and only a few exhibited high titer levels (Supplementary Fig. S1A–D). For EV-B species, the moderate and high nAb titers were frequently detected in seropositive subjects, especially in the 0 to 6 and 7 to 19 age groups, except ECHO25 (Supplementary Fig. S1E-H).

### The seroprevalence and GMT of different types of EVs in gender and district groups

No significant gender-specific difference in seroprevalence and GMT was observed for most EVs, with the exception of CV-A16, ECHO30, and EV-A71 (Table 3). In all, 20.8% of the males and 28.8% of the females showed nAbs to CV-A16 (*P* = 0.034), while 51.0% of the males and 25.7% of females showed nAbs to CV-A10 (*P* < 0.0001). The GMT of males (1:52.6) was significantly higher than that of females (1:37.2) in EV-A71-seropositive subjects (*P* = 0.005). Even though there were some minor differences, the overall results suggested that gender was not a major factor affecting most EVs. In addition, Xiamen City is a major seaport and tourist destination in southeastern China, which is divided into six districts (Supplementary Fig. S2). The distributions of seroprevalence and GMT among districts were diverse (Table 3), but in general, the seroprevalence of nAb in Haicang, Jimei, and Xiang’an were relatively high, especially against CV-A6, while for Siming district, it was lower in all types of EVs.

**Table 3:** Seroprevalence and GMT of nAb against different types of enteroviruses by gender and district

	Pathogen, seroprevalence % (GMT)							
	EV-A71	CV-A16	CV-A6	CV-A10	CV-B3	CV-B5	ECHO25	ECHO30
Gender								
Male (*n* = 255)	34.5 (52.6)	20.8 (47.4)	36.1 (48.1)	38.4 (43.1)	31.4 (100.4)	30.6 (58.0)	13.7 (32.0)	51.0 (78.0)
Female (*n* = 260)	40.7 (37.2)	28.8 (44.6)	42.7 (47.4)	46.9 (47.4)	37.7 (97.1)	33.8 (58.7)	15.0 (33.2)	25.7 (73.2)
District								
Siming (*n* = 83)	26.5 (51.3)	18.0 (33.5)	20.1 (28.3)	34.9 (43.7)	25.3 (66.1)	20.5 (48.1)	7.2 (22.6)	16.9 (45.3)
Huli (*n* = 95)	47.4 (39.1)	28.4 (45.8)	34.7 (50.8)	41.1 (41.0)	30.5 (65.5)	33.7 (55.0)	7.4 (32.0)	30.5 (105.7)
Haicang (*n* = 84)	33.3 (34.5)	29.8 (64.0)	54.8 (54.2)	42.7 (41.1)	31.0 (112.0)	35.7 (53.2)	19.1 (51.4)	29.8 (73.5)
Jimei (*n* = 82)	41.5 (44.3)	25.6 (49.1)	47.6 (47.3)	39.0 (52.7)	30.5 (147.0)	36.6 (51.2)	18.3 (29.2)	20.7 (104.4)
Xiang’an (*n* = 82)	40.2 (45.7)	29.3 (39.2)	42.7 (55.7)	57.3 (43.0)	42.7 (135.8)	32.9 (80.6)	22.0 (34.6)	24.4 (43.7)
Tong’an (*n* = 89)	36.0 (51.5)	18.1 (41.5)	37.1 (42.0)	41.6 (54.0)	47.2 (89.0)	33.7 (64.0)	13.5 (22.6)	28.1 (86.8)

*nAb* neutralizing antibody; A neutralizing titer of ≥ 1:16 was used as a threshold for seropositive;* n* the number of seropositive samples, *GMT* geometric mean titer

### Co-existence of nAbs against different types of EVs in healthy subjects

**Fig. 2:**
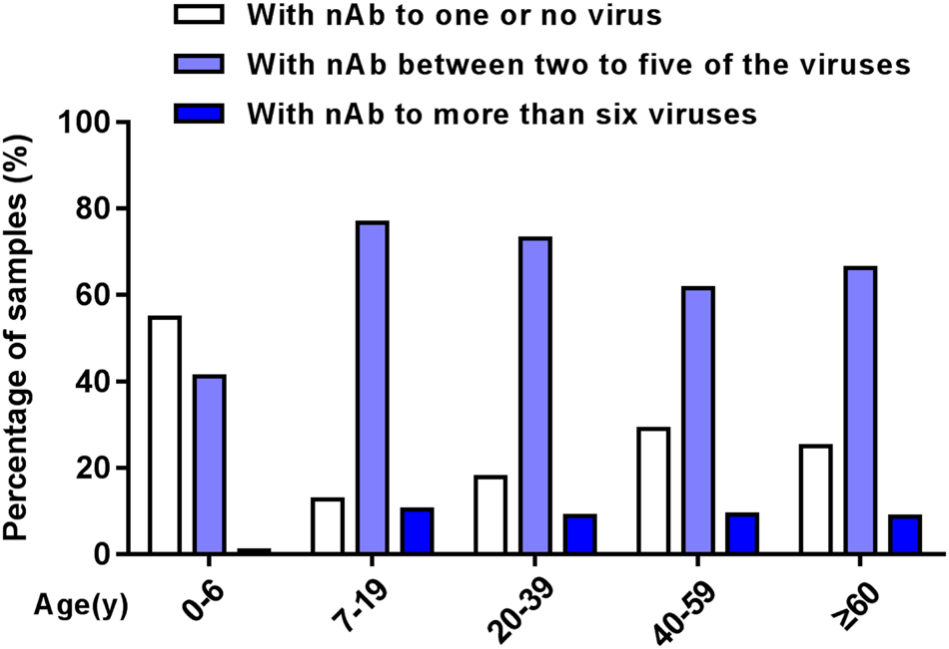
Co-existence of nAbs against different types of enterovirus in healthy subjects

**Fig. 3:**
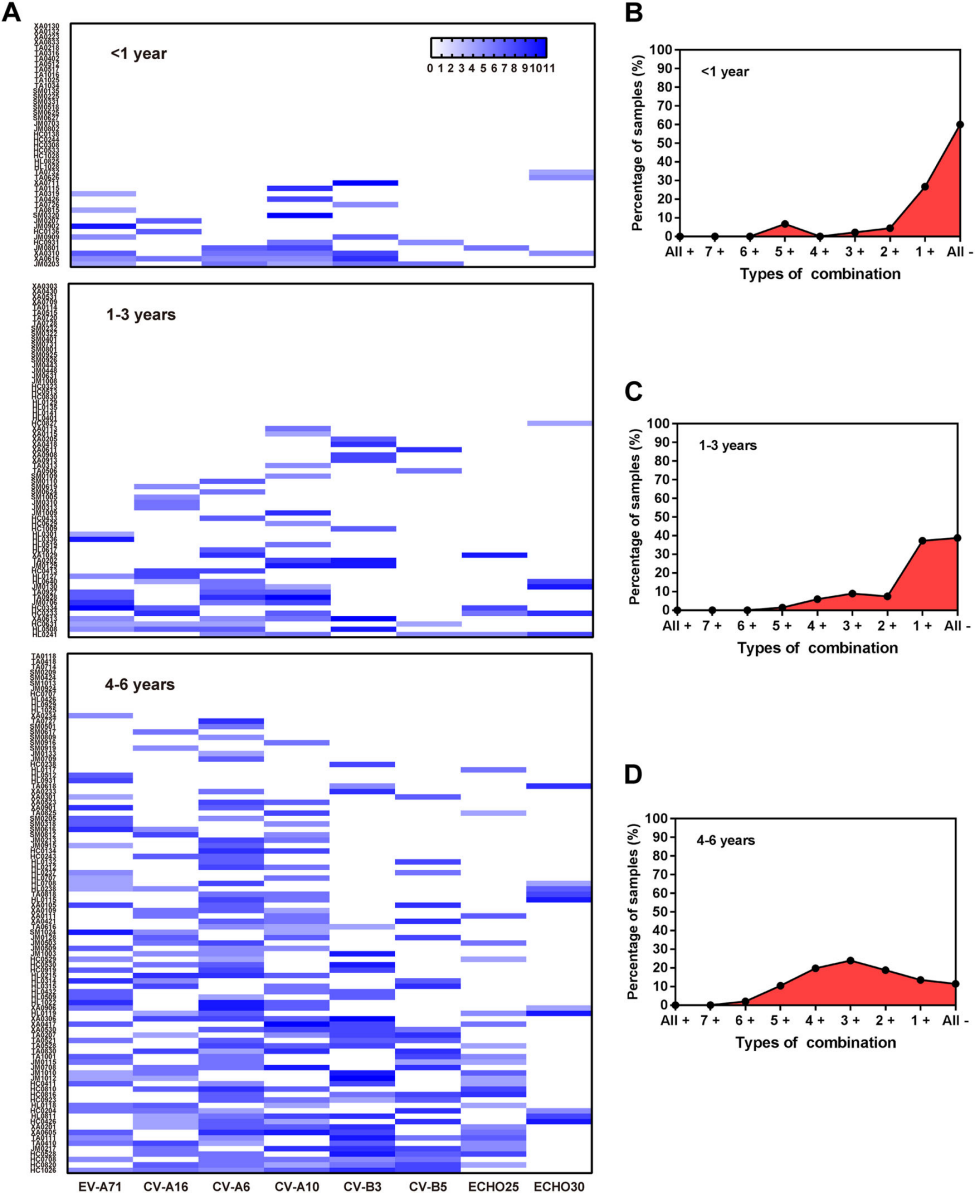
Seroprevalence and GMT of nAb against different types of enterovirus by age groups. Clustering analysis in the existence of nAb against eight types of EVs in 0–6 years age group (**a**). Co-existence of nAbs in < 1 year age group (**b**), 1–3 years age group (**c**) and 4–6 years age group (**d**). “All + ”, nAbs against all eight EVs are positive; “7 + ” means that nAb against any seven of the EVs are positive and so on; “All-”, nAbs against eight EVs are negative

The co-existence of nAbs against eight types of EVs among healthy subjects is shown in Fig. 2. Of 208 total children ≤ 6 years, 114 (54.8%) subjects were negative or positive for only 1 EV, which was significantly higher than other age groups (*P* < 0.005). Eighty-six (41.3%) children were positive for nAbs against between two to five types of virus, while more than 60% of subjects were > 6 years of age. Only a few had nAbs against more than six viruses, of which only 3.9% were in the 0 to 6 years group. Further comprehensive analysis showed that the proportion of subjects without nAb against all eight EVs was decreased with age in children significantly, ranging from 60% to 38.8% to 11.5%, respectively, in infants, toddlers, and pre-schoolers (*P* < 0.005) (Fig. 3a–d), and the pre-schoolers primarily had nAbs against 2 to 4 EVs (Fig. 3d).

## Discussion

EVs are the most common causative agents of many harmful diseases. Several EV-related molecular epidemiology studies have focused on analyzing the evolution of pathogens and classifying pathogen species, but few studies have focused on the serological survey of nAbs against EVs.The level of nAbs against human EVs reflects the history of previous infection and the specific immunity, which is important for preventing viruses and reducing the risk of disease occurrence. Here, we firstly conducted a cross-sectional serological survey of EV-A71, CV-A16, CV-A6, CV-A10, CV-B3, CV-B5, ECHO25, and ECHO30 at the same period of time in a large cohort from Xiamen City to gain comprehensive insight into the seroepidemiology of these eight EVs. Our data showed that the overall seroprevalence of the above-mentioned and high-profile EVs in the healthy population were ranged from 14.4 to 42.7%, the GMT of nAbs were ranged 32.6 to 98.6, which suggesting that a high percentage of people had been exposed to EVs.

EV-A71, CV-A16, CV-A6, and CV-A10 are belonged to EV-A species, which are the main pathogens of HFMD epidemics. HFMD is a highly infectious disease. The number of its reported cases has increased rampantly, since a large nationwide HFMD outbreak occurred in mainland China in 2008^38^. According to the national notifiable disease reporting system in China, HFMD reported cases increased from 1,156,000 in 2009 to 2,442,138 in 2016^39^. In 2016, HFMD reported cases in Xiamen were up to 8851, which had increased 28.1% from 2015 (data provided from Xiamen CDC). In this study, we found that the seroprevalence of CV-A10, CV-A6, and EV-A71 were significantly higher than CV-A16, respectively, indicating that these three viral infections have been prevalent in Xiamen in recent years, while CV-A16 infections were less common. These results meet with a recent study of the molecular epidemiology and etiology of HFMD in Xiamen City from 2008 to 2015, which indicated that EV-A71 was always one of the most predominant pathogen, and the proportion of CV-A6 was fairly high, which replaced CV-A16 as one of the most common genotypes in Xiamen City, from 2013^40^. Additionally, for CV-A10, CV-A6, and EV-A71, people aged ≤ 3 years had significantly lower levels than those > 7 years, and the higher nAb titers were observed in children, suggesting that the main pathogens of HFMD infections occurred in children ≤ 6 years.

Human EV-B species can cause not only HFMD, but the more severe outcomes. Our study analyzed the nAb levels of CV-B3, CV-B5, ECHO25, and ECHO30, revealing that these viruses were widely distributed in Xiamen, except ECHO25. By far, there has been no reports about the serological survey of CV-B5, ECHO25, and ECHO30. The seroprevalence distribution in age group of CV-B3 in our research was similar to that of a previous survey in mainland China^41^. We found that the higher nAb titers were frequently detected in CV-B3, CV-B5, and ECHO30, especially in the 0 to 6 and 7 to 19 age groups, demonstrating that these three viral infections might elicit stronger nAb responses in comparison to other EV infections.

Our data also showed that more than 60% of people > 7 years had co-existence of nAb against 2 to 5 viruses, and approximately 10% had nAb against more than six types. The data suggested that they were once vulnerable to multiple alternate infections or co-infections, due to the co-circulation of EVs. In recent years, surveillance data has indicated that EV-A71, CV-A16, CV-A6, and CV-A10 were combined in different ways, which frequently co-infected and co-circulated in HFMD outbreaks^17, 19^. Other rare combinations have also been found, such as EV-A71 and vaccine-derived polivirus (VDPV)^42^, CV-A10 and CV-B1^19^. Co-infection could enhance the severity of the clinical manifestations, and some studies have shown that co-circulation may prompt extensive genetic recombination^20, 21^.

With the increased emphasis on child care and early education, the number of nurseries and kindergartens grows every year. These child centers frequently experience EV-related disease outbreaks, especially HFMD. Our survey showed that most children were negative for nAb against EVs, especially those < 1 year of age. Significantly, among 208 children, the sorts of viruses caused by infection increased with age. Children are the most susceptible population of major EV-related illnesses, which is closely associated with their poor immunity and the range of activity. Additionally, we found that there is a wide range of linkage in seroprevalence between CV-A6 and other seven EVs, while there is nearly no linkage between ECHO30 and other EVs, in children aged 0 to 6 years (Supplementary Fig. S3A). The reason for this feature might be that CV-A6 had the highest infection rate in children, reflected by the highest seroprevalence of CV-A6 (36.1%), while the seroprevalence of ECHO30 (8.7%) was lowest among all eight EVs in the age group of 0 to 6 years (Supplementary Fig. S3B). These results indicated that CV-A6 infections were more common in children aged 0 to 6 years, while ECHO30 infections were less common. However, we also noticed that the seroprevalence of ECHO30 increased significantly in the group aged 7 to 19 years (36.0%) against the group aged 4 to 6 years (10.4%), which indicated that ECHO30 is more common in the early youth. Human EVs include more than 100 serotypes, causing the occurrence of many diseases. Currently, only three monovalent inactivated EV-A71 vaccines are commercially available, but EV-A71 vaccination cannot prevent infections caused by CV-A10, CV-A6, and other EVs. Co-infection by and co-circulation of various viruses are common. Therefore, it is urgent to develop multivalent vaccines against various viruses simultaneously, especially prevalent pathogenic strains.

In conclusion, we investigated the seroprevalence of EV-A71, CV-A16, CV-A6, CV-A10, CV-B3, CV-B5, ECHO25, and ECHO30 in a general population from Xiamen City. We analyzed the distributions of nAbs in age, gender, and district, and the correlation of prevalence between any two viruses. This is the first report detailing the seroepidemiology of eight prevalent EVs in the same population, which provides a comprehensive insight into the seroepidemiology of EVs and has implications for the development of vaccine and therapeutic drug design for EV-related disease prevention and control.

## Electronic supplementary material

Supplementary Figure S1

Supplementary Figure S2

Supplementary Figure S3
