# Supplementary figures and images for: Serological survey of neutralizing antibodies to eight major enteroviruses among healthy population
Source: Emerg Microbes Infect. 2018 Jan 10;7:2. doi: 10.1038/s41426-017-0003-z (PMC5837151; doi:10.1038/s41426-017-0003-z)

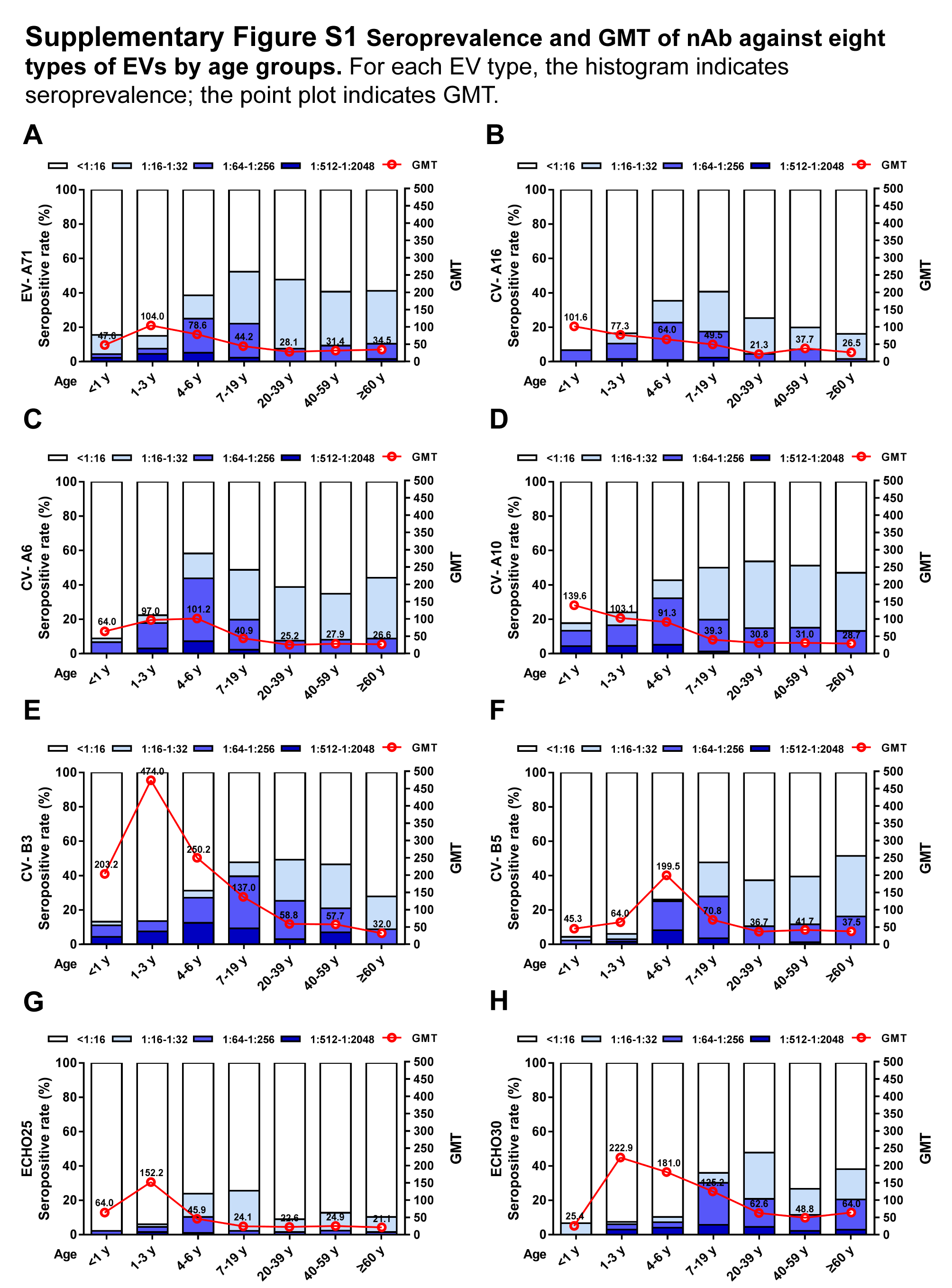

Supplement: Supplementary file 1 — Supplementary Figure S1 [file 41426_2017_3_MOESM1_ESM.tif]

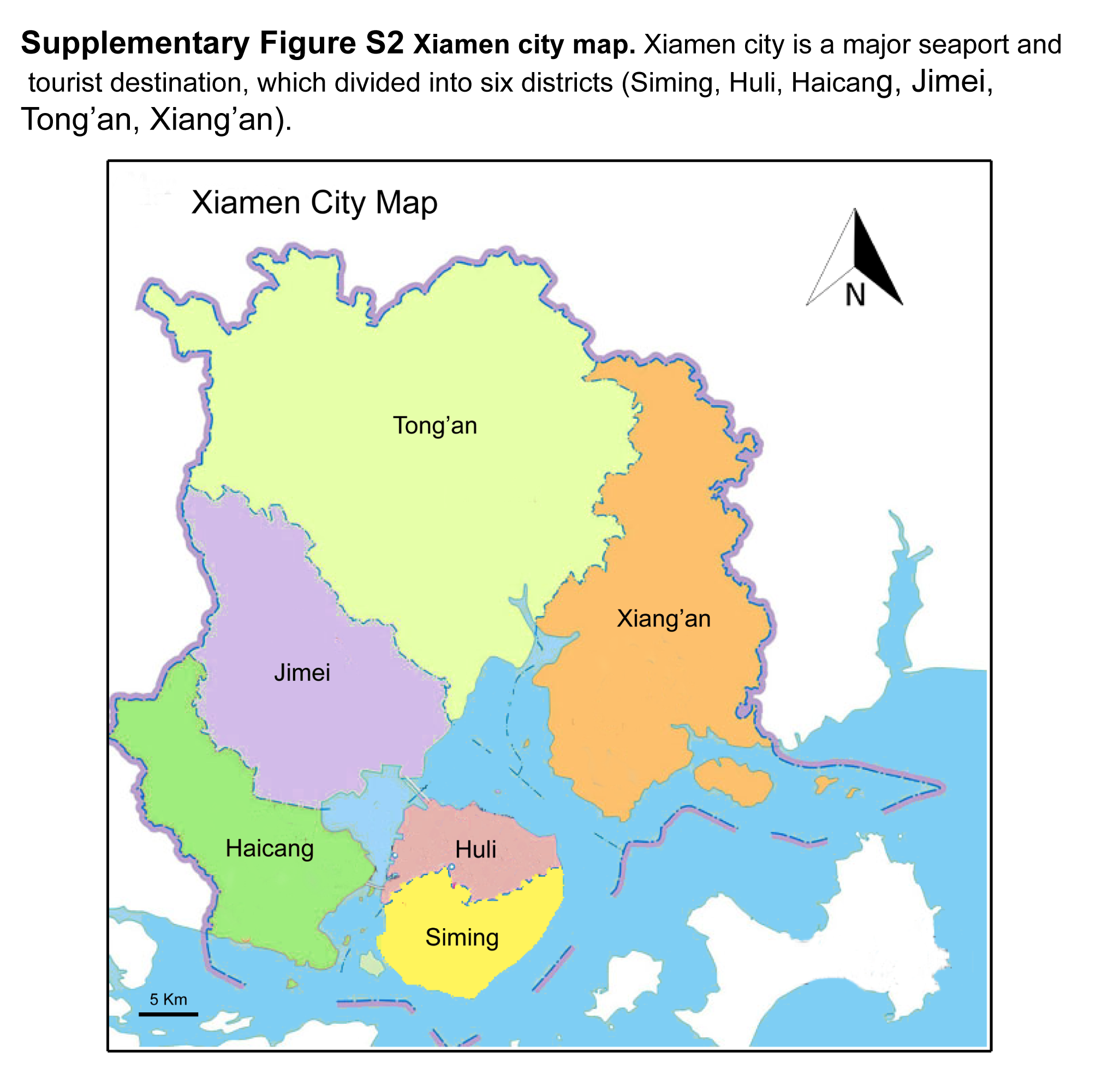

Supplement: Supplementary file 2 — Supplementary Figure S2 [file 41426_2017_3_MOESM2_ESM.tif]

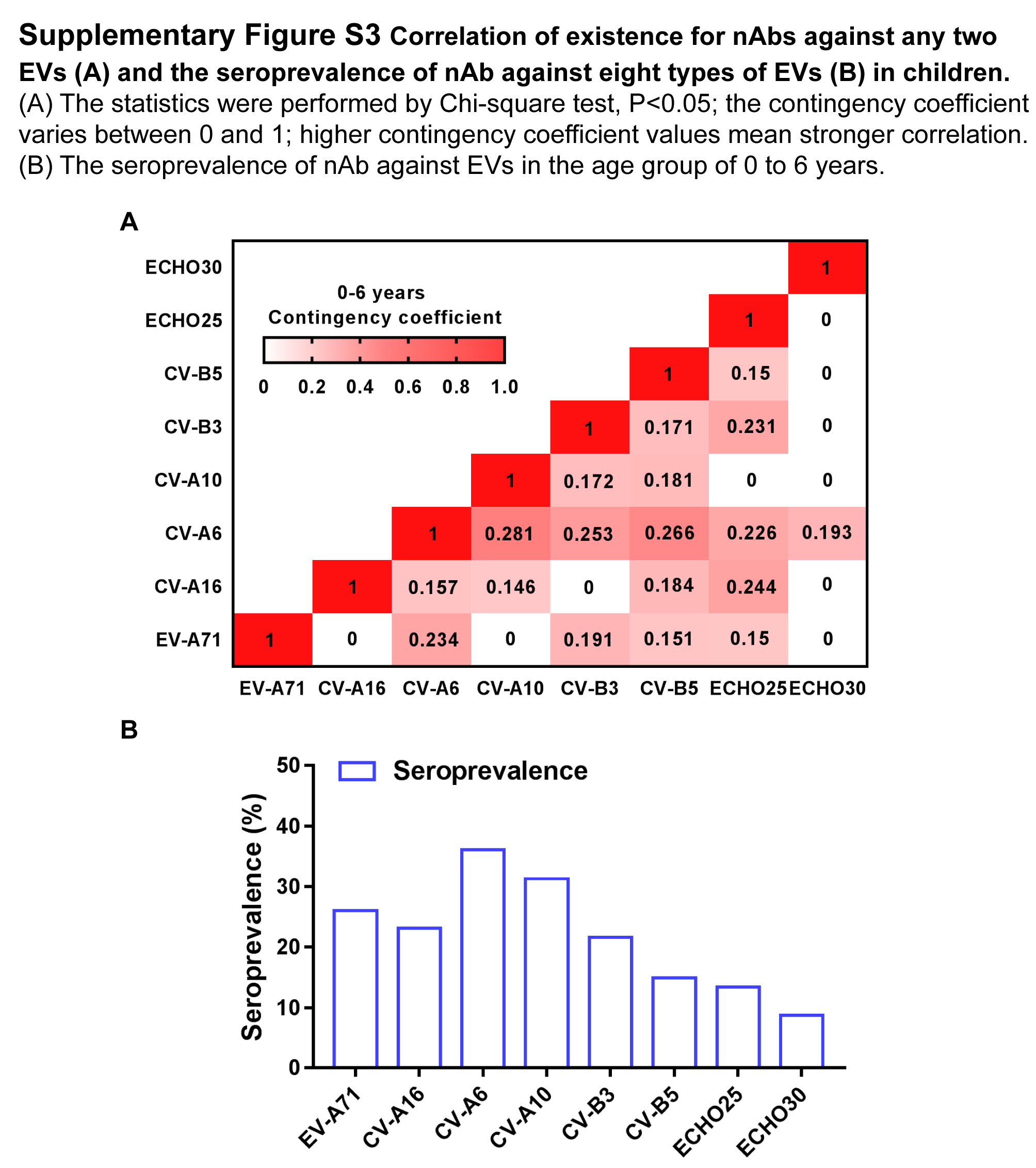

Supplement: Supplementary file 3 — Supplementary Figure S3 [file 41426_2017_3_MOESM3_ESM.tif]
